# SARC-F Is a Predictor of Longer LOS and Hospital Readmission in Hospitalized Patients after a Cardiovascular Event

**DOI:** 10.3390/nu14153154

**Published:** 2022-07-30

**Authors:** Ana Paula Trussardi Fayh, Francisco Felipe de Oliveira Guedes, Guilherme Carlos Filgueira Calado, Sandra Azevedo Queiroz, Marina Gabriely Gomes Barbosa Anselmo, Iasmin Matias de Sousa

**Affiliations:** 1 Postgraduate Program in Nutrition and Health Sciences, Health Sciences Center, Federal University of Rio Grande do Norte, Natal 59078-970, Brazil; 2 PesqClin Lab, Onofre Lopes University Hospital, Brazilian Company of Hospital Services (EBSERH), Federal University of Rio Grande do Norte, Natal 59012-570, Brazil; felipe9000fo@gmail.com (F.F.d.O.G.); guilherme.calado.700@ufrn.edu.br (G.C.F.C.); marina.anselmo.120@ufrn.edu.br (M.G.G.B.A.); 3 Postgraduate Program in Nutrition, Health Sciences Center, Federal University of Rio Grande do Norte, Natal 59078-970, Brazil; sandraa_queiroz@hotmail.com; 4 Postgraduate Program in Health Sciences, Health Sciences Center, Federal University of Rio Grande do Norte, Natal 59012-570, Brazil; iasmin_matias@hotmail.com

**Keywords:** sarcopenia, cardiovascular disease, acute myocardium infarction

## Abstract

It is already established that sarcopenia is associated with adverse outcomes; however, few studies have focused on patients who have suffered an acute cardiovascular event. The use of SARC-F, a 5-item sarcopenia screening questionnaire, in these patients remains to be investigated. We aimed to investigate whether SARC-F can predict adverse outcomes in patients admitted to a hospital with a suspected infarction. This is a 1-year prospective cohort study. During hospitalization, patients completed the SARC-F questionnaire (scores ≥ 4 considered positive for the risk of sarcopenia). Length of hospital stay (LOS), new hospital admission, myocardial infarction, and cardiovascular mortality were collected via medical records and phone interviews. In total, 180 patients were evaluated. The median age was 60.6 years; 72.3% of the participants were men, and half of the sample had comorbidities. The median SARC-F score was 1.0 (interquartile range, 0–3.0), and 21.1% of the participants screened positive. Risk of sarcopenia was independently associated with longer LOS (odds ratio, 2.34; 95% CI, 1.09–5.04; *p* = 0.030) and hospital readmission (odds ratio, 3.73; 95% CI, 1.60–8.69; *p* = 0.002). One-fifth of post-acute cardiovascular event patients in this cohort screened positive for sarcopenia using the SARC-F screening questionnaire. Positive scores were associated with a longer LOS and hospital readmission.

## 1. Introduction

Several studies have revealed an association between low muscle mass and strength and arterial stiffness, an independent predictor of cardiovascular disease (CVD) [1,2,3,4]. However, to date, little is known about the association between sarcopenia and adverse outcomes after an acute cardiovascular event.

Among acute CVD patients, the prevalence of death due to acute myocardium infarction (AMI) is high. Of all out-of-hospital cardiac arrests, AMI is responsible for almost half of deaths when considering all ages; this proportion increases progressively with age [5,6]. Additionally, with increasing age, the risk of the patient developing sarcopenia increases [7], resulting in negative effects, including falls, fractures, functional disability, enhanced hospital admission rates, reduced quality of life, and even death [8]. Some studies have also reported a relationship between muscle mass parameters and adverse outcomes in post-AMI patients [9,10] and other cardiovascular conditions [11,12]. Therefore, screening sarcopenia is pivotal in this population.

Numerous screening tools are available to screen sarcopenia. The SARC-F was developed in 2013 [13] and screens patients at risk for sarcopenia, which includes deficiencies in strength, walking, rising from a chair, climbing stairs, and experiencing falls. Each of the self-reported parameters receives a specific score (from 0 to 2), with the greatest maximum SARC-F score being 10 [10,13,14]. Although the accuracy of this questionnaire has mainly been verified in healthy people living in the community [14,15] and in patients with cancer [16,17,18], few studies have been conducted on patients with CVD. To our knowledge, no study has been conducted on post-acute cardiovascular event patients, including AMI. Therefore, the purpose of this study was to investigate whether SARC-F can predict adverse outcomes in patients admitted to a hospital with suspected infarction. We hypothesized that SARC-F scores are associated with longer length of stay (LOS) and mortality, even after adjustments for confounding factors.

## 2. Materials and Methods

### 2.1. Study Design and Sample

We conducted a single-center prospective 12-month cohort study. Adult patients (age > 20 years) of both sexes admitted to the Cardiovascular Unit of the Onofre Lopes University Hospital with symptoms of AMI between April 2019 and March 2020 were enrolled. We excluded those who had been hospitalized for 14 days or more on the date of the evaluation and stayed for more than 48 h in the Intensive Care Unit (ICU) at the beginning of hospitalization. All participants signed an informed consent form, and the study was approved by the Research Ethics Committee of Onofre Lopes University Hospital (CAAE 15610319.4.0000.5292).

The sample size calculation considered the difference in the cardiovascular event incidence between non–ST-segment–elevation myocardial infarction patients with and without sarcopenia (48% vs. 21%) [19]. Considering a power of 80%, a significance level of 5% and an additional of 20% for adjustment in multivariate analyses, the target sample size was 172 patients.

### 2.2. Procedures

**Figure 1:**
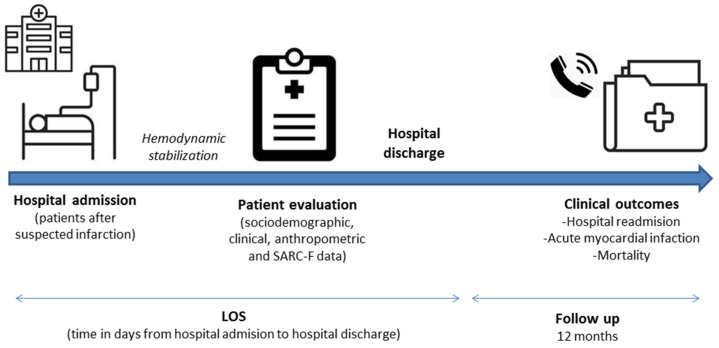
Steps of data collection. Abbreviation: LOS, length of hospital stay.

After hemodynamic stabilization, those who met the eligibility criteria were invited to participate in the study. In our institution, it is recommended that patients with indications for primary angioplasty should be admitted to the institution via hemodynamics service for percutaneous coronary intervention (PCI). According to the Brazilian Society of Cardiology Guidelines [20], after PCI is completed, the patient must return to the ICU and remain for 12 to 24 h if there are no complications. The approach and invitation occurred after the patient was discharged from the intensive care unit. Clinical data were obtained from medical records. Comorbidities were recorded and the comorbidity burden was expressed according to the Charlson Comorbidity Index (CCI) adjusted by age [21]. The LOS was recorded at the time of discharge, whereas the other outcomes were registered within 12 months of the evaluation. Figure 1 illustrates the steps of data collection.

### 2.3. Evaluations

Anthropometric data were measured by a trained technician. A digital scale (Filizola^®^) with a capacity of 200 kg was used to measure current body weight; height was measured using a stadiometer coupled to the digital scale. Body mass index (BMI, kg/m^2^) was classified according to the World Health Organization (WHO) [22]. To measure the calf circumference (CC), an inelastic tape was used (Sanny^®^, Brazil, São Paulo). For this measurement, the individuals were seated with their legs positioned at a 90° angle, and the measurement was performed in the area with the greatest circumference of the region. When the value obtained was ≤33 cm for women and ≤34 cm for men, CC measurement was considered low, according to the cutoff points proposed by Barbosa-Silva et al. [23]. Waist circumference (WC) was obtained by placing the tape in a horizontal plane at the greatest abdominal extent, based on the midpoint between the last ribs and the iliac crest. The cutoff points proposed by the WHO (>94 cm for men and >80 cm for women) were used, indicating a high risk for cardiometabolic diseases [24]. Handgrip strength (HGS) was measured using a hydraulic dynamometer (SH 5001, Sahean^®^, Changwon-City, Korea). The measurements were conducted in the arm without venous access, and the higher of the two trials was used in the analysis. Dynapenia was determined based on the reference values of the EWGSOP2 (HGS < 27 kg and <16 kg for males and females, respectively) [14].

A 5-item SARC-F questionnaire was used as a screening tool for sarcopenia [13]. The SARC-F measure includes deficiencies in strength, walking, rising from a chair, climbing stairs, and experiencing falls. Each item is scored between 0 and 2 points, yielding a total score from 0 (best) to 10 (worst). A score ≥ 4 indicates a positive risk for sarcopenia.

### 2.4. Clinical Outcomes

The primary end point was prolonged LOS (the time in days from hospital admission to hospital discharge and categorized by a median value considering the data distribution of the current sample). Secondary end points were cardiovascular events, including cardiovascular death, myocardial infarction, and hospital readmission for unstable angina. Long-term events were collected by reviewing the medical records and phone interviews and searching for the occurrence of mortality, myocardial infarction, and new hospitalization for unstable angina. The maximum follow-up time was 12 months.

### 2.5. Statistical Analysis

Data analysis was performed by the statistical package SPSS version 25.0 (IBM^®^, Chicago, IL, USA). We prespecified patients into two groups: patients at risk for sarcopenia and patients without a risk for sarcopenia. Continuous variables are presented as means ± SDs or medians with interquartile ranges. Categorical variables are presented as the number of totals (percentages). Equality of means between the two groups (with and without sarcopenia risk) was tested using the Student *t*-test or the Mann–Whitney U test for continuous variables and the Chi-square test for categorical variables. Spearman’s correlation was performed to verify the correlation between the SARC-F score and the CC and HGS. Endpoints were considered: death or the end of follow-up (12 months). Univariate and multivariate analyses were performed considering short- and long-term adverse events. Prolonged LOS, AMI, hospital readmission and death were analyzed with logistic regression, and CCI adjusted for age was considered as confounders in the analysis. A *p*-value < 0.05 was considered statistically significant for all tests.

## 3. Results

**Figure 2:**
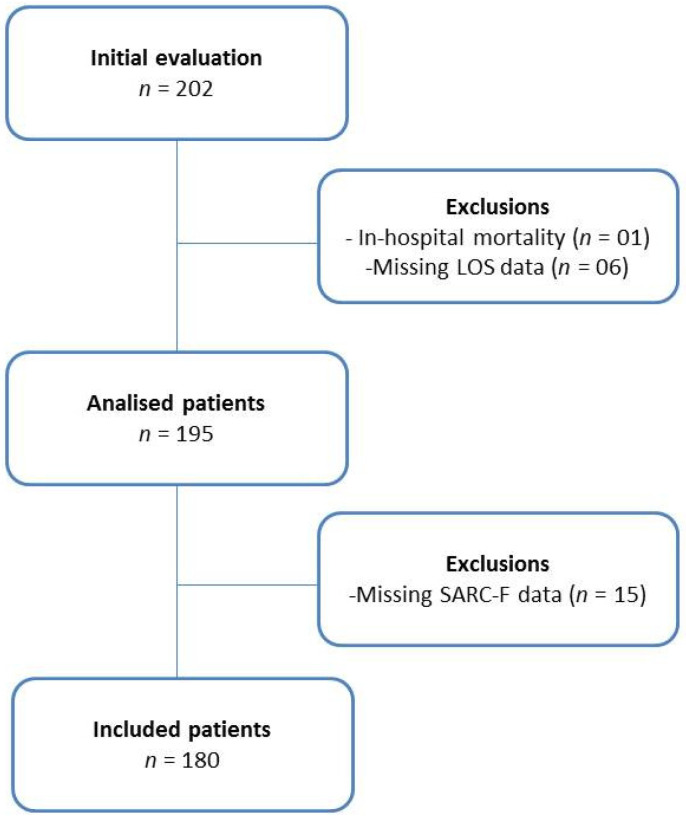
Flowchart of the cohort study.

A total of 202 patients were evaluated in this study. Of total, one died during hospitalization, six did not have data on length of stay, and 15 did not have the complete SARC-F, totaling twenty-two exclusions. Therefore, 180 participants were included; 72.8% were men. The recruitment processes are presented in Figure 2.

Table 1 presents the sociodemographic, clinical, and nutritional characteristics of the patients at baseline. The mean age corresponded to 60.6 years (56.1% older than 60 years old), and a higher frequency of older patients was observed in patients with a risk of sarcopenia (SARC-F ≥ 4).

Regarding clinical characteristics, the most frequent diagnostic was ST-segment elevation myocardial infarction; CCI indicating high risk was observed in 40.6% of the evaluated patients, with higher frequency in those with risk of sarcopenia. Around 11% have had a previous AMI and almost 30% of them are current smokers. The anthropometric assessment shows a median BMI of 26.3 kg/m^2^ with more than half of the evaluated patients having excess weight, high WC, and low CC.

The median SARC-F score was 1.0 (interquartile range, 0–3.0), and 21.1% of the participants screened positive. Patients classified at risk of sarcopenia (SARC-F ≥ 4), also presented lower CC and HGS, higher WC, and prolonged LOS compared to those without risk of sarcopenia (Table 1). Additionally, the Pearson correlation test points to a weak and moderate correlation between SARC-F scores and calf circumference (r = −0.317, *p* < 0.001) and HGS (r = −0.510, *p* < 0.001).

Table 2 presents the associations between the risk of sarcopenia (SARC-F ≥ 4) and adverse outcomes. Patients with a risk of sarcopenia had a longer LOS (LOS > 7 days) and a higher frequency of hospital readmission.

The univariate and multivariate analysis between the risk of sarcopenia and adverse outcomes demonstrated that the risk of sarcopenia was an independent predictor of prolonged LOS and hospital readmission in patients with post-acute cardiovascular events with 2.34 and 3.73 times higher odds, respectively, compared to those with no risk of sarcopenia. However, it was not a predictor of new AMI or overall death (Table 3).

## 4. Discussion

The present study first showed that SARC-F is a predictor of longer LOS and hospital readmission in patients admitted for acute cardiovascular events. This association was also significant even after adjustment for clinically important variables included in the CCI, adjusted by age.

The relationship between sarcopenia and cardiovascular risk has been discussed. Sarcopenia may promote atherogenesis due to a relative fat mass increase in response to the loss of muscle mass and the replacement of myocytes by adipocytes [25]. Santana et al. observed that the prevalence of sarcopenia and sarcopenic obesity in post-AMI patients was high (64.6% and 35.4%, respectively), but only sarcopenia was associated with thrombolysis [26]. In patients with abdominal obesity, those who tested positive in a screening test for sarcopenia had a significantly higher plasma level of B-type natriuretic peptide compared with those with a low sarcopenia score and had the poorest prognosis for cardiovascular mortality, nonfatal myocardial infarction, stroke, unstable angina, and heart failure hospitalization [27]. However, due to limited budgets and physical conditions, testing for sarcopenia (including muscle mass and strength) cannot be widely promoted in a hospital setting. For this reason, a simple method for screening for sarcopenia is needed in clinical practice.

SARC-F is one of the most common screening tools for sarcopenia. On the other hand, previous reports have indicated a low to moderate sensitivity and high specificity of SARC-F in the diagnosis of the risk of sarcopenia [14,28]. Considering that hospitalized patients have several physical limitations during bed-rest situations, the use of SARC-F as a tool for screening sarcopenia risk could be highlighted in these studies. Poor utility of the SARC-F tool in hospitalized patients related to this condition, having physical limitations unrelated to sarcopenia status, is probably acquired during hospitalization [29]. Therefore, it is important to recognize that the participants’ sarcopenia status could be associated with their LOS in the hospital, affecting their ability to perform the physical aptitudes that SARC-F evaluates.

SARC-F was developed based on the elderly population, and its applications in other populations may not be appropriate. However, in recent years, studies using SARC-F as screening of sarcopenia in different populations have been published, such as orthopedic [16], cancer [30,31], gastrointestinal [32] and type 2 diabetes patients [33], regardless of age or hospitalization status. Kurita et al. [30] examined the accuracy of SARC-F ≥ 4 in orthopedic patients and reported a sensitivity and specificity of 41.7% and 68.5%, respectively, indicating that the specificity was comparable to that of the SARC-F ≥ 4 for community-dwelling older adults. Ishida et al. [28] observed that SARC-F ≥ 4 is suitable as a screening tool for sarcopenia in hospitalized older adults (*n* = 1689). The sensitivity and specificity of SARC-F ≥ 4 for sarcopenia and possible sarcopenia were 49.1–51.3% and 73.9–81.2%, respectively. Thus, SARC-F seems to be very useful for the assessment of sarcopenia, although the sensitivity of SARC-F for sarcopenia is low.

We observed weak to moderate correlations between the SARC-F score questionnaire and the parameters of muscle mass and physical function evaluated in the appendicular regions (arms and legs). This is an expected result since the SARC-F questions are related to physical abilities involving muscle strength. In this context, Barbosa-Silva et al. [34] proposed a modified version of the SARC-F, including an anthropometric measurement as a marker of muscle mass (CC) with the aim of improving the performance of the original for screening (SARC-CalF score). Some studies have already shown that both versions have acceptable agreement for screening sarcopenia in different populations [18,32,35].

In line with previous studies, we showed that high scores on the SARC-F questionnaire were associated with a longer LOS and hospital readmission in this sample of inpatients. However, no association between sarcopenia risk and 12-month mortality was observed. To date, the literature on this issue is scarce and divergent. In a sample of 132 patients admitted to the hospital with non–ST-segment–elevation myocardial infarction, Matsumoto et al. [19] observed that muscle wasting (low muscle mass index measured by computed tomography) was a predictor of cardiovascular events, including cardiovascular deaths, non-fatal myocardial infarction, or non-fatal stroke. However, the median follow-up period for this study was 2.4 years, which is longer than the present study. Sato et al. [9] also reported that a low appendicular skeletal muscle index assessed by DXA is independently associated with poor outcomes in 387 patients with ST-segment–elevation myocardial infarction. Muscle wasting is a significant medical issue in patients with cardiovascular disease and is positively affected by adequate intervention, such as rehabilitation and nutritional treatment [36,37]. It is important to highlight that SARC-F includes questions related to muscle strength and function—not including muscle mass measures. In the “hierarchy of loss in physical function”, a decline in muscle strength and function is first observed, leading to a decline in muscle mass [38].

The present study has some limitations. The single-center design limits the generalization of the results. Since the SARC-F questionnaire was applied only at the baseline, we did not have other clinical and sociodemographic information that could also be predictors of mortality during a year of follow-up, which may have affected the results of our study. We also did not have the date of death, so we cannot carry out the Cox regression analysis to verify the association between the risk of sarcopenia and its components and mortality. Finally, due to the descriptive design of this study, our results serve as an initial exploration of the SARC-F tool in cardiac patients, and further work is needed on larger, more diverse samples with close follow-up. As a strength, the present study findings contribute to providing important information for clinical practitioners because they emphasize the risk of sarcopenia weakness to identify adverse outcome predictions in patients admitted to the hospital for an acute myocardium infarct.

In conclusion, our hypothesis was partially confirmed. SARC-F was a predictor of longer LOS and hospital readmission in adult patients admitted to a hospital for AMI. Early detection and intervention of sarcopenia are crucial for hospitalized patients. Thus, SARC-F appears to be an appropriate screening tool for adverse outcome risks in hospitalized post-cardiac events.

## Figures and Tables

**Table 1:** Sociodemographic, clinical and nutritional characteristics of patients after acute cardiovascular event at baseline (*n* = 180).

Characteristics	Total *n* = 180 (100%)	SARC-F < 4 *n* = 142 (78.9%)	SARC-F ≥ 4 *n* = 38 (21.1%)	*p*-Value
Age (years)	60.6 ± 12.7	59.0 ± 12.5	66.3 ± 11.7	**<0.001** ^1^
>60 years	101 (56.1%)	74 (52.1%)	27 (71.1%)	**0.043** ^2^
Males	131 (72.8%)	118 (83.1%)	13 (34.2%)	**<0.001** ^2^
Current smoker	54 (30.0%)	38 (26.8%)	16 (42.1%)	0.077 ^2^
Diagnostic				0.823 ^3^
Angina	15 (8.3%)	12 (8.5%)	3 (7.9%)	
STEMI	134 (74.7%)	104 (73.2%)	30 (78.9%)	
NSTEMI	31 (17.2%)	26 (18.3%)	5 (13.2%)	
CCI	2.0 (1.0; 3.0)	2.0 (1.0; 3.0)	3.0 (2.0; 4.5)	**<0.001** ^4^
CCI ≥ 2	73 (40.6%)	49 (34.5%)	24 (63.2%)	**0.002** ^2^
Previous AMI	19 (10.6%)	16 (11.3%)	3 (8.1%)	0.768 ^3^
BMI (kg/m^2^)	26.3 (23.8; 29.2)	26.6 (24.0; 29.7)	26.0 (22.3; 28.5)	0.171 ^4^
Overweight (BMI ≥ 25 kg/m^2^)	113 (63.1%)	92 (64.8%)	21 (56.8%)	0.445 ^2^
Obesity (≥30.0 kg/m^2^)	39 (21.7%)	33 (23.2%)	6 (16.2%)	0.385 ^2^
CC (cm)	33.6 (31.0; 36.0)	34.2 (31.7; 36.5)	32.0 (29.5; 33.7)	**0.001** ^4^
Low CC	99 (55.0%)	70 (49.3%)	29 (76.3%)	**0.003** ^2^
WC (cm)	98.0 (90.0; 103.9)	97.1 (88.8; 103.6)	99.0 (91.6; 104.0)	0.425 ^4^
High WC	124 (68.9%)	93 (65.5%)	31 (83.8%)	**0.032** ^2^
Handgrip Strenght (HGS, kg/F)	26.0 (16.0; 32.0)	28.0 (22.0; 34.0)	13.0 (3.5; 22.50)	**<0.001** ^4^
Low HGS	83 (46.1)	59 (41.5%)	24 (63.2%)	**0.018** ^2^
LOS (days)	7.0 (5.0; 14.3)	7.0 (5.0; 11.0)	10.0 (7.0; 22.5)	**0.008** ^4^

AMI, acute myocardium infarction; BMI, body mass index; CC, calf circumference; CCI, Charlson Comorbidity Index; STEMI, ST segment elevation myocardial infarction; NSTEMI, non-ST segment elevation myocardial infarction; WC, waist circumference. ^1^ Student’s *t*-test; ^2^ Chi-squared test; ^3^ Fisher’s Exact test; ^4^ Mann Whitney U Test. Bold values means a statistically significant *p*-value.

**Table 2:** Risk of sarcopenia and short- and long-term adverse events in patients after an acute cardiovascular event (*n* = 180).

Adverse Events	SARC-F < 4 *n* = 142 (78.9%)	SARC-F ≥ 4 *n* = 38 (21.1%)	*p*-Value
Longer length of hospital stay (*n* = 86)	61 (43.0%)	25 (65.8%)	**0.017**
New AMI (*n* = 16)	11 (8.3%)	5 (15.6%)	0.314
Hospital readmission (*n* = 36)	21 (15.8%)	15 (44.1%)	**0.001**
Overall mortality (*n* = 14)	9 (6.7%)	5 (15.2%)	0.155

AMI, acute myocardium infarction. Bold values means a statistically significant *p*-value.

**Table 3:** Univariate and multivariate analyses of the association between the risk of sarcopenia and adverse outcomes in patients after an acute cardiovascular event (*n* = 180).

SARC-F	Univariate OR (95% CI)	*p*-Value	Multivariate ^1^ OR (95% CI)	*p*-Value
Longer length of hospital stay (*n* = 86)	2.55 (1.21–5.40)	**0.014**	2.34 (1.09–5.04)	**0.030**
New AMI (*n* = 16)	2.05 (0.66–6.40)	0.214	1.27 (0.38–4.20)	0.695
Hospital readmission (*n* = 36)	4.21 (1.85–9.58)	**0.001**	3.73 (1.60–8.69)	**0.002**
Overall mortality (*n* = 14)	2.48 (0.77–7.97)	0.127	1.88 (0.55–6.44)	0.316

AMI, acute myocardium infarction. Logistic Regression, ^1^ Multivariate analysis adjusted by Charlson Comorbidity Index (dichotomized). Bold values means a statistically significant *p*-value.

## Data Availability

Data available on request due to ethical and privacy restrictions. The data presented in this study are available on request from the corresponding author. The data provided by the volunteers are not publicly available due to privacy/ethical restrictions.
